# Effectiveness of tafenoquine and primaquine for radical cure of *Plasmodium vivax*: a meeting report from dissemination of results of the EFFORT trial to stakeholders in Pakistan

**DOI:** 10.1186/s12936-025-05636-8

**Published:** 2025-11-11

**Authors:** Bushra Qurashi, Shan-e-Zehra Zaidi, Muhammad Mushtaque, Ahmed Faisal, Zeeshan Haroon, Baseer Agha, Naseer Hammal, Abdul Hameed, Ali Raza, Umair Ali, Asia Khan, Fareeha Abdul Jabbar, Zainab Rafeeq Adam, Tariq Mehmood, Ric N. Price, Najia Ghanchi, Kamala Thriemer, M. Asim Beg

**Affiliations:** 1 Department of Pathology and Laboratory Medicine, Aga Khan University, Karachi, Pakistan; 2 Vector-Borne Diseases Program, Directorate General Health Services Sindh, Hyderabad, Pakistan; 3 Directorate Malaria Control Program (DoMC), Islamabad, Pakistan; 4 Khyber Medical University, Khyber Pakhtunkhwa, Pakistan; 5 Vector-Borne Diseases Program, Baluchistan, Pakistan; 6 Common Management Unit (AIDS, TB & Malaria), Ministry of Health, Baluchistan, Pakistan; 7 Global and Tropical Health Division, Menzies School of Health Research, Charles Darwin University, Darwin, Australia; 8 Mahidol Oxford Research Unit, Faculty of Tropical Medicine, Mahidol University, Bangkok, Thailand; 9 Center for Tropical Medicine and Global Health, Nuffield Department of Medicine, University of Oxford, Oxford, UK

**Keywords:** Pakistan, Malaria vivax, Radical cure, Tafenoquine, Primaquine

## Abstract

*Plasmodium vivax* remains the predominant cause of malaria in Pakistan, accounting for approximately 85% of confirmed cases. The recurrent nature of *P. vivax*, driven by dormant liver-stage hypnozoites, poses a major obstacle to malaria control. Pakistan currently uses a 14-day low-dose primaquine regimen (3.5 mg/kg total dose) without routine G6PD testing, a strategy limited by poor adherence and suboptimal efficacy. In 2024, the World Health Organization (WHO) recommended high-dose primaquine (7 mg/kg) or single-dose tafenoquine (300 mg) as more effective alternatives, but evidence on their programmatic implementation remains limited. The EFFORT clinical trial evaluated the safety and effectiveness of 7-day-high-dose primaquine and single-dose tafenoquine compared to the standard 14-day-low-dose regimen. Conducted across four endemic countries, including Pakistan, the trial found both regimens to be well tolerated and effective in preventing relapse. In Pakistan, the added benefit of high-dose primaquine over the standard regimen was modest but consistent with findings from other South Asian countries. The findings of EFFORT highlight significant benefits of tafenoquine in *P. vivax* endemic settings beyond those currently endorsed in the 2024 WHO guidelines. A national dissemination meeting was held at the Aga Khan University hospital Karachi on 10th April 2025 organized by Aga Khan University and Menzies School of Health Research. The meeting brought together national and provincial stakeholders to review study results and explore implications for radical cure policy and implementation in Pakistan. Key themes included policy alignment, phased implementation of tafenoquine and G6PD testing, planning and funding constraints, private sector engagement, and the importance of sustained dialogue between researchers and malaria program leaders.

## Background

Despite progress in malaria control, *Plasmodium vivax* remains the predominant cause of malaria in Pakistan, accounting for approximately 85% of the more than 400,000 confirmed malaria cases in 2022 [1]. Over 95% of Pakistan’s malaria case burden is concentrated in the provinces of Sindh (49.4%), Balochistan (37.9%) and Khyber Pakhtunkhwa (8.0%), where seasonal transmission patterns and limited access to effective treatment hinder malaria control efforts [2]. In the aftermath of the devastating floods in mid-2022, the country experienced an unprecedented surge in malaria cases, with a fivefold increase of cases to more than 3.4 million , of which nearly two-thirds were attributed to *P. vivax* [3]. Accelerated by this increase, the National Malaria Control Programme revised its strategic plan to place greater emphasis on *P. vivax* as the dominant parasite species. This included the development of a *P. vivax* readiness and implementation plan aimed at strengthening surveillance, diagnosis, and radical cure strategies [4].

A key challenge in controlling *P. vivax* is its ability to form dormant liver-stage parasites (hypnozoites) that can reactivate weeks or months after the initial infection leading to relapse. Effective treatment of *P. vivax* malaria requires a two-pronged therapeutic approach: a blood-stage schizontocidal medication such chloroquine or an artemisinin-based combination therapy (ACT) to clear the acute infection and a hypnozoiticidal agent to eliminate liver-stage parasites and prevent relapse. The only drugs currently available for this purpose are the 8-aminoquinolines primaquine and tafenoquine. Both primaquine and tafenoquine can cause severe haemolysis in individuals with glucose-6-phosphate dehydrogenase (G6PD) deficiency, an inherited enzymatic disorder affecting red blood cell stability. G6PD deficiency is common across malaria-endemic regions and is estimated to affect between 3 and 7% of the population in Pakistan, with regional variation [5].

In the absence of routine G6PD testing, many malaria-endemic countries have adopted conservative treatment strategies, using low-dose primaquine (0.25 mg/kg daily for 14 days) to minimize the risk of drug-induced haemolysis. In Pakistan, current national guidelines recommend this regimen without prior G6PD screening [2].

This approach faces two major limitations. First, adherence to a prolonged 14-day unsupervised regimen is often poor, leading to decreased effectiveness in routine clinical settings [6, 7]. Second, the low total dose is suboptimal and higher doses (7 mg/kg total dose) have been shown to reduce the risk of recurrence by 50% in almost all geographic settings [8]. In recognition of this, the 2024 World Health Organization (WHO) guidelines now recommend higher-dose primaquine regimens (7 mg/kg total over 7 or 14 days) or single-dose tafenoquine (300 mg) as effective alternatives [9]. Tafenoquine, which has now been licensed in ten endemic countries [10], is a single-dose treatment that may overcome the adherence challenges inherent to multi-day primaquine regimens. In its licensing trials, tafenoquine has been benchmarked against low-dose primaquine and no direct random comparison with high-dose primaquine were conducted to date. Furthermore, the effectiveness of these new radical cure regimens under routine programmatic conditions, particularly when treatment is unsupervised, remained uncertain.

To address the limited evidence on the effectiveness of these new radical cure regimens, the EFFORT trial (NCT04411836) was conducted to evaluate the safety and efficacy of 7-day-high-dose primaquine (1 mg/kg/day, 7 mg/kg total dose) and single-dose tafenoquine (300 mg) compared to the standard 14-day-low-dose primaquine treatment (0.25 mg/kg/day, 3.5 mg/kg total dose). The study was conducted as a multi-center randomized controlled trial with study sites in Cambodia, Ethiopia, Indonesia, and Pakistan.

Detailed findings of the trial are presented elsewhere [11]. In brief, the trial showed that the 7-day-high-dose primaquine and tafenoquine regimens were well tolerated and both treatments led to a significant reduction of recurrences compared to patients treated with 14-day-low-dose primaquine. In the Pakistan context, the added benefit of high-dose primaquine over low-dose primaquine appeared relatively modest, consistent with findings from other data from South Asia [12]. The trial findings support the geographic extension of the current tafenoquine recommendation with chloroquine to high burden areas such as Pakistan.

## Meeting details

Organized by the Aga Khan University in collaboration with the Menzies School of Health Research, the dissemination meeting for the EFFORT study results in Pakistan took place on10th April 2025, in Aga Khan University Hospital Karachi. The objective of the meeting was to present findings from the EFFORT trial and discuss their implications for the case management of *P. vivax* malaria in Pakistan.

The event was attended by a wide range of participants, including 17 representatives from the Sindh and Federal Health Departments of Pakistan as well as 7 public health experts from Aga Khan University. In addition, 5 district health officers, 15 laboratory personnel, and 5 field staff from the study site were also present.

The agenda included four main components: (i) an opening session with welcome remarks, (ii) presentation of EFFORT safety and effectiveness results for the overall trial and Pakistan specific data followed by (iii) a question and answer session and (iv) a panel discussion. The panel included decision-makers from both federal and provincial levels (Table 1) and was guided by a set of structured discussion questions highlighted in Table 2.

**Table 1:** List of panelists

1	Prof. Kamala Thriemer	Menzies School of Health Research, Darwin, Australia
2	Prof. M. Asim Beg	Aga Khan University, Karachi, Pakistan
3	Dr. Muhammad Mushtaque	Directorate General Health Services Sindh, Hyderabad, Pakistan
4	Dr. Zeeshan Haroon	Khyber Medical University, Khyber Pakhtunkhwa, Pakistan
5	Dr. Baseer Agha	Vector-Borne Diseases, Baluchistan, Pakistan
6	Mr. Naseer Hammal	Vector-Borne Diseases program, Directorate General Health Services Baluchistan, Quetta, Pakistan
7	Mr. Abdul Hameed	Directorate General Health Services Sindh, Hyderabad, Pakistan

Panel Moderator: Dr. Najia Ghanchi—Associate Professor & V Chair, Aga Khan University

**Table 2:** Questions posed to panelists for the panel discussions

What are the key challenges in implementing tafenoquine as a standard treatment for malaria in Pakistan especially in hard-to-reach areas?
How can we address safety concerns associated with the use of tafenoquine in the Pakistani population?
Do you think we need more data to rollout tafenoquine in Pakistan if yes what could be the role of malaria control program to generate more data?
Can partnerships with international organizations help subsidize the costs of testing and treatment in malaria endemic areas? How can we make it sustainable?
How can Pakistan integrate G6PD testing into its national malaria control and treatment programs?
What is the current infrastructure in Pakistan for diagnosing G6PD deficiency?
How can government and health organizations facilitate reducing costs and make these tests accessible to populations in malaria high-risk zones?
What role can public awareness campaigns play in educating the population and healthcare workers about the importance of G6PD testing before starting tafenoquine for malaria radical cure?

Throughout the meeting, notes were taken and collated to present key discussion points. The following themes emerged from the discussion: (i) policy coherence and the risk of misalignment: Proposed shift to artemether-lumefantrine, (ii) operational and implementation challenges: the need for a stepwise approach, (iii) funding and planning constrain in a fragmented health system, (iv) private sector exclusion from planning and policy processes, (v) strengthening the dialogues between research national malaria control programme.

## Policy coherence and the risk of misalignment: proposed shift to artemether-lumefantrine

There is growing concern within Pakistan’s malaria control community regarding a potential change of the first-line schizontocidal treatment for *P. vivax* from chloroquine to artemether-lumefantrine. Artemether-lumefantrine is a widely accepted ACT, and is safe and effective treatment for both *Plasmodium falciparum* and *P. vivax* [13], in co-endemic regions a universal policy for uncomplicated malaria due to all species of malaria has significant logistical benefits [14]. However, there are some concerns that this proposed shift may be driven by procurement and logistical convenience, particularly the easier accessibility of artemether-lumefantrine compared to chloroquine from global suppliers. There is currently no domestic production of chloroquine in Pakistan. Importantly this strategic shift may jeopardize Pakistan’s plans to introduce tafenoquine. Currently tafenoquine is only licensed in combination with chloroquine. This restriction was based on data from Indonesia where tafenoquine in combination with dihydroartemisinin-piperaquine, another ACT, showed low efficacy [15]. While results from the EFFORT study at the sites in Cambodia and Indonesia challenge this understanding [11], more data with different artemisinin-based combinations, including artemether-lumefantrine, are needed. In the meantime, a shift to artemether-lumefantrine would likely protract the introduction of tafenoquine in Pakistan.

## Operational and implementation challenges—a stepwise approach

A key concern raised by stakeholders was whether the infrastructure and systems in Pakistan are equipped to support the safe use of tafenoquine, which requires testing for G6PD prior to administration. To address these challenges, participants discussed a phased or stepwise rollout strategy that would allow the health system to gradually build operational experience, foster community acceptance and increase confidence among health workers, policy makers and patients.

This approach is consistent with implementation strategies used in other endemic countries. Brazil, the first malaria-endemic country to approve tafenoquine for *P. vivax* radical cure, adopted a phased introduction model. Rollout began in settings with well-established diagnostic capacity, allowing health authorities to monitor uptake, identify challenges early, and adjust strategies accordingly [16–19]. Participants in the Pakistan meeting similarly emphasized the importance of initiating tafenoquine implementation in high-capacity areas, using early experience to inform broader scale-up. Many countries have begun rolling out G6PD testing as a first preparatory step, with the goal of enabling future implementation of tafenoquine and higher-dose primaquine regimens [20]. This allows programmes to familiarize health workers with testing procedures, address logistical bottlenecks, and build the foundation for safe radical cure delivery [21, 22].

Importantly, Pakistan has already initiated a G6PD testing pilot study supported by Medicines for Malaria Venture (MMV) through the Asia–Pacific Malaria Elimination Network (APMEN) in nine high-burden districts, with training delivered to provincial program managers, laboratory staff, and clinicians in December 2023 in Islamabad [23]. This pilot is designed explicitly as a preparatory step for future introduction of both tafenoquine and high-dose primaquine regimens, allowing the health system to practice testing procedures, resolve supply chain issues, and build provider proficiency before expanding radical cure at scale. This phased, capacity-building approach mirrors global best practices and was recognized by stakeholders as a critical pathway for safe and sustainable uptake of tafenoquine in Pakistan.

## Funding and planning challenges in a fragmented system

The introduction of tafenoquine in Pakistan faces substantial funding challenges because of the additional cost for G6PD testing, compounded by uneven health system and decentralized governance structure. A recurring theme raised by stakeholders was the need to ensure consistent and equitable access to financing across provinces to support the introduction of new tools such as tafenoquine. While the Global Fund provides critical support for malaria control in several provinces, others fall outside the scope of current funding agreements [24]. This variation in coverage presents challenges for national-level planning and raises concerns about potential disparities in access to diagnostics and treatment innovations.

Participants emphasized the importance of engaging more closely with the Global Fund—not only for sustained funding, but also to ensure alignment with the operational research and implementation activities already underway in the country [25]. Planning and coordination are particularly complex in Pakistan, where provinces are responsible for program delivery, yet often depend on federal or donor-led forecasting for policy and funding. These structural dynamics make it challenging to implement national scale interventions particularly for new interventions requiring aligned resources and technical guidance. Without deliberate coordination there is a risk that some provinces, particularly those with stronger institutional capacity and donor engagement may move ahead more quickly, while others may lag behind due to governmental or financial constrains [26]. Participants emphasized that a phased rollout must be guided by a national strategy targeting areas with highest caseloads first to avoid deepening existing inequities and to ensure that all provinces ultimately benefit from access to new radical cure tools.

## Private sector exclusion from planning and policy processes

In Pakistan, the private healthcare sector plays an important role in malaria case management. Approximately 80% of outpatient visits occur in private facilities, including clinics, pharmacies, and informal providers, especially in rural and peri-urban areas where public services are limited [27]. The actual malaria burden could be considered four to five times higher, since nearly 70–80% of the population resorts to the private sector for treatment; therefore, those malaria cases are not notified [28]. Despite this substantial patient volume, private providers are often excluded from formal malaria treatment (including radical cure treatment) and surveillance systems, limiting the reach and impact of national malaria programmes. Better coordination between the public and private sector has been planned by the malaria control programme in the next five years to improve reporting for diagnosis and treatment at private tertiary and secondary healthcare facilities in Pakistan [29].

As stakeholders discussed the phased introduction of tafenoquine, they emphasized that this rollout must not be confined to the public sector alone. A phased approach offers an opportunity to pilot engagement models with registered private clinics in selected districts, including provision of G6PD testing, access to tafenoquine, and participation in pharmacovigilance systems. Experience from successful malaria programmes in other countries has shown that structured commitment with the private sector can improve case management and treatment [30].

Participants also noted that stronger advocacy and policy mechanisms are needed to elevate the role of the private sector in malaria decision-making processes. At present, most discussions about tafenoquine introduction and associated operational planning occur within public sector or academic spaces, with limited input from private health providers.

## Strengthening the dialogue between researcher and the malaria control program

A key theme emerging from the meeting was the importance of strengthening the dialogue between researchers and the national and provincial malaria control programme. Stakeholders from both the public sector and academia expressed appreciation for early access to nationally generated research findings. Timely dissemination of trial results enabled real-time engagement with new evidence and supported a discussion on the implications for both strategic planning and field level implementation.

There was broad support for fostering closer collaboration between researchers and decision-makers at both provincial and federal levels. Participants called for the strengthening of already available mechanisms such as joint technical working groups and the establishment of evidence translation platforms through which researchers and implementers can review evidence, adjust implementation strategies, and co-develop solutions. Embedding these collaborations within routine malaria programme governance was seen as essential for building a culture of shared learning.

## Conclusion

The EFFORT trial provides key evidence to support the use of tafenoquine Pakistan. The stakeholder meeting provided a timely platform for reviewing the findings of the EFFORT study and discussing their relevance for Pakistan’s malaria control strategy.

While both single dose tafenoquine and 7-day-high-dose primaquine hold significant promise in improving adherence and reducing relapse, their successful implementation will depend on addressing key operational barriers, most notably the introduction of G6PD testing and health system strengthening.

The trial findings highlight the importance of the ongoing revisions of Pakistan’s national malaria treatment protocols to include new radical cure approaches, equally important will be translating these policy shifts into action at the provincial and district levels, supported by phased implementation, coordinated planning, and stakeholder engagement including the private sector.

## Data Availability

No datasets were generated or analysed during the current study.

## Abbreviations

ACT: Artemisinin-based Combination Therapy
APMEN: Asia-Pacific Malaria Elimination Network
G6PD: Glucose-6-phosphate dehydrogenase
MMV: Medicines for Malaria Venture
WHO: World Health Organization
